# Health Data for New York City Overview: Advancing Health Equity through Policy-Relevant Collaborative Research

**DOI:** 10.1007/s11524-021-00587-2

**Published:** 2021-11-19

**Authors:** Michele Calvo, Elizabeth Kelman, L. Hannah Gould, R. Charon Gwynn, Lisa M. Bates, Marivel Davila, Francesca Gany, Mary Huynh, David Siscovick

**Affiliations:** 1 New York Academy of Medicine, New York City, NY USA; 2 Division of Epidemiology, New York City Department of Health and Mental Hygiene, New York City, NY USA; 3 Columbia University Mailman School of Public Health, New York City, USA; 4 Center for Health Equity and Community Wellness, New York City Department of Health and Mental Hygiene, New York City, NY USA; 5 Memorial Sloan Kettering Cancer Center, New York City, NY USA; 6 Weill Cornell Medical College, New York City, NY USA

## Introduction

Actionable policy-relevant research is critical to improving population health and advancing health equity, as research provides an evidence base for the development of effective policies and programs. For academic researchers with an interest in addressing the upstream social and structural causes of health inequities, conducting this type of research remains a challenge. To maximize the utility of research findings, academic researchers need to collaborate with researchers with applied public health experience, including government and other stakeholders, but may not have the time and knowledge needed to navigate complex government bureaucracy. To influence policy and program development, academic researchers also must understand the broader, applied context, along with the skills needed to communicate relevant findings to decision-makers.

While large city health departments collect vast amounts of data and make efforts to expand access to those data, there remain real and perceived barriers to the effective and efficient use of these data for maximum policy and programmatic impact [1, 2]. Applied public health researchers within a local health department may have limited access to expertise in advanced analytic methods or familiarity with agency data resources outside of their individual program. Health department employees often do not have adequate time for research, analysis, and writing, although research may be of interest and helpful to elevate careers.

There are an array of initiatives that aim to promote multi-institutional, multi-sectoral collaboration; expand access to available population health data; or provide mentorship for early- and mid-career academic researchers [3, 4]. We hypothesized that a program that combined these core elements in the context of New York City (NYC), which has a diverse population, large urban health department, and many academic institutions, would provide a unique opportunity to advance actionable health equity-focused research.

In this issue of the *Journal of Urban Health*, we describe the development of Health Data for New York City (HD4NYC), which aims to advance policy-relevant, health equity-focused research by combining multi-institutional and interdisciplinary collaboration, access to data, and mentorship. We also describe select initial research products emerging from this new, unique program.

## The Health Data for New York City Program

HD4NYC was launched by the New York Academy of Medicine (NYAM) and the New York City Department of Health and Mental Hygiene (Health Department) in March 2019, with funding from the Robert Wood Johnson Foundation. HD4NYC builds on the long-standing commitment of the Health Department to provide public access to its many data sources as well as existing collaborations between the Health Department and academic researchers. The goals of HD4NYC are to (a) produce actionable, policy-relevant research that will advance health equity and (b) advance the careers of a diverse group of early- and mid-career investigators from NYC metropolitan academic institutions and the Health Department.

HD4NYC is based on the research working group model that has been shown to foster multi-institutional collaboration, high impact research, and career development of early- and mid-career investigators in other settings, such as the Cardiovascular Health Study (CHS) and the the Cohorts for Heart and Aging Research in Genomic Epidemiology (CHARGE) [5, 6].

Our program differs in important ways from prior applications of the working group model:

1. HD4NYC promotes collaboration between academic and applied public health researchers.
2. HD4NYC facilitates access to a breadth of data sources, including Health Department survey and surveillance data.
3. HD4NYC focuses on the complex and interrelated social, economic, and environmental drivers and consequences of health inequities.
4. HD4NYC supports the translation of research findings into policy change.

The Health Department and NYAM provide shared leadership for the program and dedicated program staff, including a program coordinator at NYAM and a program coordinator and data manager at the Health Department. These roles support accessing and analyzing data, navigating the Heath Department, connecting to external stakeholders, project management, and other logistical and administrative needs. Each working group is co-led by senior mentors from the Health Department and an academic institution.

### Recruitment

We used multiple strategies to recruit a diverse pool of academic and Health Department investigators. We recruited investigators and co-leads within the Health Department via a variety of agency networks. Potential working group topic areas were identified based on applicants’ interests, agency priorities, and availability of Health Department data. After identifying possible working group topics, we invited leaders in health equity to nominate early career academic investigators, conducted an environmental scan, and sent email blasts. We assessed applicants on the following attributes: demonstrated commitment to health equity, policy-relevant research, and team science; analytical skills; and alignment of interest with the initial themes. For academic investigators, we also assessed publication and funding productivity. For Health Department investigators, we considered how HD4NYC would facilitate career growth and skill development, as well as the applicant’s program to ensure representation from across the agency.

We accepted 19 academic investigators and two academic co-leads into HD4NYC. These researchers represent 14 academic institutions in the NYC metropolitan area and have diverse expertise, including public health, medicine, nursing, engineering, and psychology. We accepted ten Health Department investigators and two co-leads from eight Health Department divisions into HD4NYC. After 2 years, 90% of the investigators remain engaged in the program.

### Working Groups

We ultimately established two working groups: one focused on birth and childhood equity and the other focused on the health of marginalized populations. Each working group meets monthly to review research progress, obtain peer and co-lead feedback on research, and stay abreast of program goals. Working groups were further divided based on initial subthemes of interest, with three in each working group (six total). Subgroups meet on a biweekly or weekly basis to collaborate on research projects. The initial subgroups focused on the following topics: maternal/infant health, children’s environmental health, health of children from immigrant families, policy evaluation, stigma and discrimination, and criminal legal system-involved populations.

### Data Access

HD4NYC facilitated orientation to Health Department data in several ways, such as creating one-page summaries of select Health Department surveys and surveillance systems; connecting researchers to key staff that manage datasets aligned with HD4NYC subthemes; and summarizing data access procedures. We supported data access by registering academic investigators as Health Department volunteers, allowing for access to Health Department buildings and computers. We also offered software trainings, arranged for remote data access, and facilitated the completion of Data Use Agreements. Finally, our data manager ran descriptive statistics to guide development of research questions and analytic plans.

### Training and Mentorship

To support the professional development of investigators and enhance the quality and policy relevance of HD4NYC research, we hosted quarterly seminars that enabled investigators to interact with national and local health equity leaders, including prominent health equity researchers, directors of local community-based organizations, and representatives from potential funding mechanisms. Mentorship is a central component of the program for both Health Department and academic investigators. HD4NYC co-leads facilitate working group meetings, guide the development of research questions, review and provide feedback on project proposals and research products, and provide as-needed one-on-one or small group mentorship.

### Stakeholder Engagement

We invited Health Department staff whose work aligned with investigator interests to join working group and subgroup meetings to inform project development. In some instances, they joined the projects as collaborators. While not explicit in the original program model, we quickly recognized the need to involve other governmental and community-based stakeholders in the collaborative research process. To start, we held a half-day stakeholder workshop, attended by invited representatives from community-based organizations and other city agencies whose work aligned with the initial six projects. HD4NYC subgroups have continued engagement in a variety of ways, including holding subsequent feedback sessions to interpret preliminary results, guest-presenting on coalition webinars, involving stakeholders in weekly project meetings, inviting stakeholders to co-author publications, and sharing findings in ways useful to community-based stakeholders (e.g., one-pagers).

### Evaluation

We used a combination of surveys and focus groups to assess the appropriateness, effectiveness, efficiency, and impact of program components, and to identify gaps in the program and priorities for improving HD4NYC. Overall, the working group model and program components were reported as valuable. HD4NYC has produced publications of peer-reviewed manuscripts, a grant-funded ancillary study, and ongoing partnerships with community and governmental stakeholders. Reported limitations included a lack of funding for academic investigators and adequate resources and time for equitable community stakeholder engagement.

## Overview of HD4NYC Research

We now provide an overview of the subset of HD4NYC research projects included in this special section. The enclosed papers address important upstream determinants of health including criminal justice, tobacco control, environmental exposures, and access to healthcare.

### Criminal Legal System

There is mounting evidence that criminal legal system policies and practices differentially impact some racial and ethnic groups, such as Black and Latinx communities; these differences may contribute to health inequities [7]. **Thompson, Baquero, and colleagues** examined experiences of policing and criminal legal system policies and their associations with physical, mental, and behavioral health outcomes (poor physical and mental health, serious psychological distress, and binge drinking). The study found that those stopped by the police, abused by the police, and those experiencing discrimination reported higher levels of adverse health outcomes, and these associations were most commonly observed among Black NYC residents and residents ages 24–44. This study strengthens the body of literature around health and policing by examining validated and comprehensive measures of health at the individual level in a representative sample of NYC residents. These findings may inform ongoing policy discussions in NYC and nationally regarding policing and criminal legal system reforms [8].

### Menthol Cigarette Ban

Well-timed, rigorous policy evaluations can provide evidence to make equity-informed policy decisions and inform the prioritization of resources. **Li and colleagues** constructed a micro-simulation model to predict cardiovascular disease outcomes and related healthcare cost savings if a menthol ban was implemented in NYC. The model projected that a menthol ban policy would result in substantial reductions in cases of myocardial infarction and stroke among adult smokers in NYC overall, and subgroup analyses suggested even greater reductions in adverse cardiovascular outcomes among women, specifically Black women [9].

### Maternal Healthcare

There is growing evidence that limited access to healthcare for populations that are marginalized is an important determinant of health. In NYC, half of all births are to immigrant mothers, yet there is little research available on immigrant maternal healthcare utilization. **Maru, Glenn, and colleagues** collaborated closely with community stakeholders to examine utilization of maternal healthcare services before, during, and after pregnancy among immigrant mothers in NYC from 2016 to 2018. The authors found lower utilization of preconception healthcare and dental cleaning during pregnancy among immigrant women, particularly among immigrants that recently arrived to the USA. The authors also found substantial variation by region of origin. These findings can help target efforts to address inequities in maternal healthcare access. The investigators are collaborating with government and community stakeholders to translate their findings into policy and practice [10].

### Socioenvironmental Contributors to Childhood Asthma

While many environmental exposures and socioeconomic factors are known to increase the risk of asthma exacerbations – the leading cause of pediatric hospitalizations and emergency department visits in NYC – they are not commonly studied together. **Bajwa, Khan, and colleagues** used cluster analysis that grouped children based on socioenvironmental and housing factors at individual and neighborhood levels to better understand the major patterns of these exposures and their relationship to asthma exacerbations. The authors found three neighborhood level factors that predominantly drove cluster formation and differed in asthma outcomes: renting homes, high-density buildings, and older buildings. This information may help target interventions to reduce asthma disparities among children in the city [11].

HD4NYC research projects not included in this issue also address important determinants and consequences of health inequities, such as bullying among LGBTQ youth of color and trends in health service utilization among children from immigrant families.

## Summary

We have successfully developed and launched a unique public-private partnership that combines collaboration, access to data, and mentoring, using a working group model, to advance health equity research and promote the careers of early- and mid-career academic and applied public health researchers. Working groups focused on marginalized populations and birth and childhood equity established six subtheme groups that have conducted rigorous and impactful research utilizing 10 datasets from the Health Department and other agencies, most in collaboration with community stakeholders. The reports included in this issue reflect the initial products of four of the subtheme groups. In April 2021, the Robert Wood Johnson Foundation awarded a grant to continue HD4NYC for a third year. During this time, we have formed a new working group on COVID-19 health inequities and are focusing on strengthening community engagement, translating research findings for policy change, and sustaining collaborative research.
